# MiR-4448 is involved in deltamethrin resistance by targeting *CYP4H31* in *Culex pipiens pallens*

**DOI:** 10.1186/s13071-021-04665-x

**Published:** 2021-03-16

**Authors:** Xixi Li, Shengli Hu, Haitao Yin, Hongbo Zhang, Dan Zhou, Yan Sun, Lei Ma, Bo Shen, Changliang Zhu

**Affiliations:** Department of Pathogen Biology, Nanjing Medical University, Nanjing, Jiangsu 211166 People’s Republic of China

**Keywords:** MicroRNA, Cytochrome P450, Insecticide resistance, CDC bottle, Mosquito

## Abstract

**Background:**

*Culex pipiens* (*Cx. pipiens*) complex, which acts as a vector of viruses and is widespread and abundant worldwide, including West Nile virus, Japanese encephalitis virus, and Sindbis virus, can cause serious vector-borne diseases affecting human health. Unfortunately, mosquitoes have developed deltamethrin resistance because of its long-term overuse, representing a major challenge to mosquito control. Understanding the molecular regulatory mechanisms of resistance is vital to control mosquitoes. MicroRNAs (miRNAs) are short non-coding RNAs that have been demonstrated to be important regulators of gene expression across a wide variety of organisms, which might function in mosquito deltamethrin resistance. In the present study, we aimed to investigate the regulatory functions of miR-4448 and *CYP4H31* in the formation of insecticidal resistance in mosquito *Culex pipiens pallens.*

**Methods:**

We used quantitative real-time reverse transcription PCR to measure miR-4448 and *CYP4H31* (encoding a cytochrome P450) expression levels. The regulatory functions of miR-4448 and *CYP4H31* were assessed using dual-luciferase reporter assays. Then, oral feeding, RNA interference, and the American Centers for Disease Control and Prevention bottle bioassay were used to determine miR-4448’s association with deltamethrin resistance by targeting *CYP4H31* *in vivo*. Cell Counting Kit-8 (CCK-8) was also used to detect the viability of pIB/V5-His-*CYP4H31*-transfected C6/36 cells after deltamethrin treatment *in vitro*.

**Results:**

MiR-4448 was downregulated in the deltamethrin-resistant strain (DR strain), whereas *CYP4H31* was downregulated in deltamethrin-susceptible strain. *CYP4H31* expression was downregulated by miR-4448 recognizing and binding to its 3′ untranslated region. Functional verification experiments showed that miR-4448 overexpression resulted in lower expression of *CYP4H31*. The mortality of miR-4448 mimic-injected DR strain mosquitoes was higher than that of the controls. CCK-8 assays showed that *CYP4H31* decreased cellular resistance to deltamethrin *in vitro* and the mortality of the DR strain increased when *CYP4H31* was knocked down *in vivo*.

**Conclusions:**

In mosquitoes, miR-4448 participates in deltamethrin resistance by targeting *CYP4H31*. The results of the present study increase our understanding of deltamethrin resistance mechanisms.
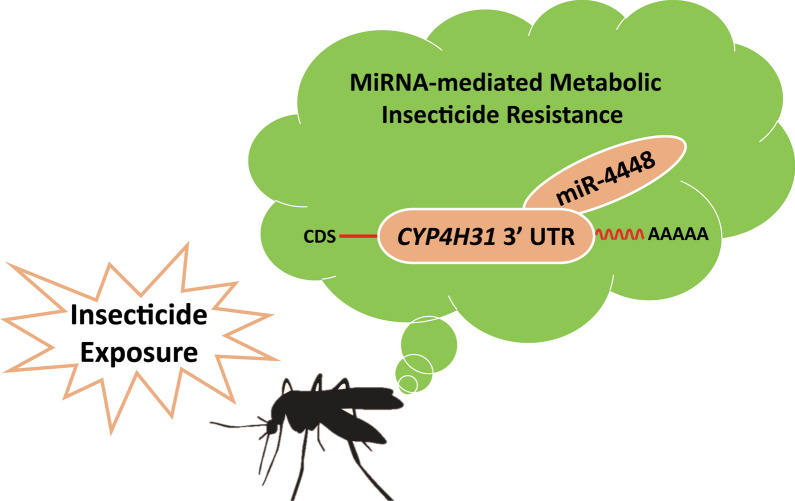

## Background

Many serious neglected tropical diseases (NTDs) diseases, including Zika, yellow fever, dengue fever, West Nile fever, and malaria, are transmitted by mosquitoes [1–4]. Globalization and international travel have contributed to transnational pathogen and vector dispersion, representing a key threat for millions of people and animals worldwide [5, 6]. Among these vectors, *Culex pipiens* complex is widely distributed worldwide and plays important roles in the transmission of many human diseases [7]. Over the past 2 decades, insecticide-based approaches to control mosquito vectors have substantially reduced the prevalence of mosquito borne diseases, including malaria [8]. Deltamethrin is a pyrethroid insecticide that is commonly used and recommended for in-home insect control because of its high efficiency, broad spectrum, and relatively low toxicity to humans [9, 10]. Unfortunately, mosquito resistance to insecticides has developed because of their long-term heavy use [11].

Studies showed that the development of insecticide resistance in mosquitoes is a complex and heritable evolutionary phenomenon, involving multiple genes and mechanisms [12]. Improving our understanding of the molecular mechanisms of insecticide resistance would allow the formulation of novel strategies to minimize and prevent resistance development, thus controlling mosquitoes [13]. To date, research has largely focused on identifying insecticide resistance-related genes and has found that the evolution of insecticide resistance is mostly induced by changes in the expression of cytochrome P450 genes [14]. In resistant mosquitoes, several P450 genes are upregulated and overexpressed [15–17]. However, the regulatory mechanisms of P450s remain largely unknown.

MicroRNAs (miRNAs) are a class of evolutionarily highly conserved non-coding small RNAs. They are 21–22 nucleotides in size and are widely distributed in eukaryotic cells [18]. MiRNAs negatively regulate gene expression at the mRNA level by recognizing and binding to 3′ untranslated regions (UTRs) [19]. They could lead to degradation of the target mRNA or inhibition of its translation, resulting in decreased production of the protein [20]. Similar to those in other animals and insects, mosquito miRNAs (22–24 nt) degrade their target mRNAs to regulate the host-pathogen interactions, metabolism, development, and insecticide resistance; however, miRNAs’ precise role in deltamethrin resistance remains mostly unknown [21].

The present study aimed to use previously identified differentially expressed miRNAs between a deltamethrin-resistant (DR-) strain and a deltamethrin-susceptible (DS-) strain of *Culex pipiens pallens* [22] to investigate miRNAs related to mosquito deltamethrin resistance. Quantitative real-time reverse transcription PCR (qRT-PCR) was used to verify the expression level of miR-4448 in DS and DR strain mosquitoes. Bioinformatic predictions and dual-luciferase assays were used to identify the potential target of miR-4448. Next, RNA interference (RNAi) was performed for miR-4448 and its target *CYP4H31* using oral feeding or microinjection, and then the mortality of mosquitoes was detected using CDC bottle bioassays *in vivo*. Cell Counting Kit-8 (CCK-8) was also applied to verify the viability of C6/36 cells transfected with *CYP4H31* *in vitro*. These experiments helped us to determine whether miR-4448 is involved in deltamethrin resistance by targeting *CYP4H31* in *Culex pipiens pallens*.

## Materials and methods

### Insects

In this study, we used two strains of *Culex pipiens pallens* with different resistance levels to deltamethrin mosquitoes. The DS strain of *Culex pipiens pallens* used in the present study was obtained from Ji Nan University and maintained in the laboratory with a constant light/dark cycle (14:10 h) at 28 °C and 70–80% relative humidity. Adults were provided with 5% (w/v) sterilized sugar [glucose (5 g, GHTECH, Guangdong, China) was dissolved in 100 ml of deionized water and autoclaved] on a Scotch-Brite™ sponge wick (3 M, Shanghai, China) *ad libitum*. Mosquitoes were fed on mouse blood to reproduce the next generation. The DR strain was selected from the DS strain and was maintained via treatment with deltamethrin at the 50% lethal concentration (LC_50_) of each generation (*G*_n_). There were 4000 larvae for screening in each pool (three pools/*G*_n_). For the DS and DR strains, the LC_50_ values were 0.04 and 8.5 mg/l, respectively. Deltamethrin (technical, 99.0%) was a gift from Jiangsu Provincial Center for Disease Control and Prevention (Jiangsu, China). Procedures for blood-feeding with mice in our laboratory were monitored by The National Science and Technology of China and People’s Government of Jiangsu Province Animal Care and Use Committee and Institutional Review Board (no. IACUC-1812047).

### Genomic DNA extraction, pre-miR-4448 amplification, and cloning

Genomic DNA (gDNA) was extracted from 3-day post-eclosion (3 d PE) female adult mosquitoes (*N* = 1) using a MiniBEST Universal Genomic DNA Extraction Kit, version 5.0 (Takara, Dalian, China), following the manufacturer’s instructions. gDNA quantity and quality were checked using a Thermo Scientific™ NanoDrop 2000 instrument (Thermo Fisher Scientific, Waltham, MA, USA).

Using the gDNA as a template, PCR was performed using primers (Table 1) designed according the *Cx*. *pipiens pallens* pre-miRNA sequence with the following conditions: 94 °C for 5 min; followed by 35 cycles of 94 °C for 30 s, 56 °C for 30 s, and 72 °C for 10 s; and a final extension step at 72 °C for 10 min. The PCR products were subjected to electrophoresis through a 2.0% agarose gel. A PCR fragment of around 80 bp was isolated from the gel and purified using a MiniBEST Agarose Gel DNA Extraction Kit version 4.0 (Takara) and then cloned into vector pMD 19-T (Takara). Ten microliters of the resultant plasmid was transferred into 100 μl of *Escherichia coli* One Shot^®^ TOP10 Competent Cells (Invitrogen, Carlsbad, CA, USA) for amplification [23]. Colonies were selected and analyzed using PCR and sequencing.

**Table 1:** Oligonucleotide sequences of miR-4448, U6, *CYP4H31*, and *β-actin* used for PCR and vector constructs in the present study

Name	Forward (5′–3′)	Reverse (5′–3′)
pre-miR-4448	GCTCGCACCACAACCCCG	AAGCGAGAATCATACCCCTAGACCA
miR-4448 (RT-stem ring)	CTCAACTGGTGTCGTGGAGTCGGCAATTCAGTTGAGCATACCCC	
miR-4448	ACACTCCAGCTGGGGGCTCGATGGTCTAGG	TGGTGTCGTGGAGTCG
U6	GCTTCGGCTGGACATATACTAAAAT	GAACGCTTCACGATTTTGCG
*CYP4H31*	ACTTTGATGGCGTTGGATAGC	AATCCCGCAAGAGGACTGAC
*β-actin*	AGCGTGAACTGACGGCTCTTG	ACTCGTCGTACTCCTGCTTGG
*CYP4H31* 3′ UTR-WT	CGAGCTCAAACCTGTTGATATTTTACTGGCA	CCAAGCTTTTTTTGCGCTCGATGGTTT
*CYP4H31* 3′ UTR-Δ	CGAGCTCAAACCTGTTGATATTTTACTGGCA	CCAAGCTTTTTTTGCCCACCAAGGTTT
pIB/V5-His-*CYP4H31*	GGACTAGTGAGATGGAAATGCTGATTGAGATCGTACTGG	CCGCTCGAGCGGTTTCGCGCCATAATCTTCA

### Identifying the miR-4448 precursor and the potential target of miR-4448

First, the miR-4448 precursor (pre-miR-4448) sequence was identified to ensure that the miRNA actually existed in *Cx. pipiens pallens* rather than being a sequencing artifact. We obtained a 90-bp pre-miR-4448 nucleotide sequence (GCTCGCACCACAACCCCGAATCACCGCGAGCGTACCGCCACTCCAGCACTCATGGCAC**GGCTCGATGGTCTAGGGGTATG**ATTCTCGCTT) by sequencing. The miR-4448 sequence is shown in bold. To identify the putative gene targets of miR-4448, we used 3′ UTR sequences from the *Cx. quinquefasciatus* genome in the RNAhybrid target prediction program [24]. We focused on the CYP family genes that participate in the regulation insecticidal resistance of mosquitoes, and only *CYP4H31* was identified as a potential target of miR-4448. To assess the conservation of the 3′ UTR, we amplified the 3′ UTR from *Cx. pipiens pallens.* The 3′ UTR sequence of *CYP4H31* in *Cx. pipiens pallens* was 100% identical with that from *Cx. quinquefasciatus*. Then, qRT-PCR was used to detect the expression levels of miR-4448 and *CYP4H31* in the DS and DR strains.

### Quantitative real-time reverse transcription PCR (qRT-PCR) analyses

At 3 d PE, DS and DR strain female adult mosquitoes (*N* = 10) were subjected to total RNA extraction using the RNAiso Plus reagent (Takara). The total RNA purity and concentration were checked using a NanoDrop spectrophotometer. The cDNA was synthesized from 1 μg of total RNA using a PrimeScript RT reagent Kit (Takara) and PrimeScript™ RT Master Mix (Takara) according to the manufacturer’s protocol. The cDNA was diluted 1:10, and 4 μl of the diluted cDNA solution was used as template for quantitative real-time PCR (qPCR) using the Power SYBR Green PCR Master Mix (Applied Biosystems, Foster City, CA, USA). PCR was performed in a 20-μl reaction mix containing 10 pmol of forward and reverse PCR primers [designed using Primer Premier 6.0 software (PREMIER Biosoft International, San Francisco, CA, USA)] for miR-4448 and *CYP4H31* (Table 1). MiR-4448 expression was measured using the Stem-loop RT-PCR method [25] with the following reaction conditions: 50 °C for 2 min and 95 °C for 10 min; 40 cycles of 95 °C for 15 s and 60 °C for 1 min, followed by melting-curve analysis on an ABI Prism 7300 real-time PCR Instrument (Applied Biosystems). The relative expression level of miR-4448 was normalized to the internal control U6 small nuclear (U6), and the *CYP4H31* expression level was normalized to that of *β-actin* from the DS and DR strains. The DS strain expression level was designated as 1. For each experiment, RNA from three biological replicates was used, and PCR amplification of each cDNA sample was performed in triplicate. The 2^−ΔΔCt^ method was used to calculate the relative expression levels [26].

### pMIR-REPORT vector construction, cell culture, and dual-luciferase reporter assay

We identified the region of the *CYP4H31* 3′ UTR that included the complementary sequences predicted to bind miR-4448. To mutate this region, the binding site complementary region (AUCGAGC) was replaced by UUGGUGG (3′ UTR-Δ). Two pairs of primers were designed according to the transcript sequences from *Cx. quinquefasciatus* to amplify the wild-type (WT) 3′ UTR and 3′ UTR-Δ of *CYP4H31* (Table 1). Luciferase constructs were made by amplifying and sequencing the *Cx. quinquefasciatus* putative target 3′ UTR-WT/3′ UTR-Δ sequence of the *CYP4H31* mRNA (containing the putative seed region of the miR-4448 binding sites) and using the T/A cloning method to insert them into the *HindIII* and *XbaI* sites located downstream of the *Renilla* translational stop codon within the pMIR-REPORT miRNA Expression Reporter Vector (Promega, Madison, WI, USA) [17].

At 48 h after transfection of the pMIR-REPORT constructs, assays were performed using the dual-luciferase reporter assay system (Promega). 293T cells were cultured in Dulbecco’s modified Eagle's medium (DMEM; Gibco, Grand Island, NY, USA) supplemented with 10% (v/v) fetal calf serum (FCS; Gibco) in a 5% CO_2_-humidified incubator at 37 °C [18]. Then, 6 × 10^4^ cells/well in 2.5 ml of complete growth medium was seeded and incubated in a 6-well plate for 24 h until they reached > 80% confluency. Then, 6 ng of pMIR-REPORT-UTR-WT or pMIR-REPORT- UTR-Δ treated with 6 µl of miR-4448 mimic and miRNA negative control (NC1) (GenePharma, Shanghai, China) along with 6 ng of PGL4.7 (Promega) was cotransfected using the FuGENE HD transfection reagent (Promega). Vector PGL4.7, which constitutively expresses *Renilla* luciferase, was cotransfected as an internal control to correct for differences in the efficiency of transfection and harvest between the groups. In each sample, *Renilla* luciferase was normalized using *Firefly* luciferase expression [19]. An M200 microplate fluorescence reader (Tecan, Lyon, France) was used to detect the luciferase activity. Cells were treated in triplicate, and the transfections were repeated three times.

### Oral feeding

For the oral feeding experiments, all the materials (e.g. water, glucose, and sponges) were treated with diethyl pyrocarbonate (DEPC; Sangon Biotech, Shanghai, China) to remove RNase. In each cage, DR strain pupae (*N* = 120) of *Cx. pipiens pallens* were collected in a plastic cup. The post-eclosion (PE) mosquitoes were starved for 12 h. The 12-h PE adults of the blank group (WT) were treated with 5% glucose water, while the negative control group (NC1) was given the miRNA mimic control dissolved in 5% glucose water, at a final dose of 100 nmol/l. The experimental group (miR-4448 mimic) was parallelly supplied with the miR-4448 mimic (100 nmol/l). At 48 h after treatment, RNA was extracted from female adult mosquitoes to validate the expression of miR-4448 and its target gene *CYP4H31*. The miR-4448 mimic and miRNA control mimic were obtained from GenePharma (Table 2).

**Table 2:** List of the miR-4448 mimic, miRNA control mimic (NC1), si*-CYP4H31*, and control siRNA (NC2) sequences used for RNAi in *Culex pipiens pallens*

Name	Sense (5′–3′)	Antisense (5′–3′)
miR-4448	GGCUCGAUGGUCUAGGGGUAUG	UACCCCUAGACCAUCGAGCCUU
miRNA control mimic (NC1)	UUCUCCGAACGUGUCACGUTT	ACGUGACACGUUCGGAGAATT
si*-CYP4H31*	GGGCAAAGAUUCGACAAAUTT	AUUUGUCGAAUCUUUGCCCTT
Control siRNA (NC2)	UUCUCCGAACGUGUCACGUTT	ACGUGACACGUUCGGAGAATT

### Microinjection of miR-4448 mimic and *CYP4H31* siRNA (si-*CYP4H31*)

Microinjections were conducted using a Nanoject III aspirator tube assembly (cat. no. 3-000-207, Drummond Scientific Co., Broomall, PA, USA) fitted with a needle puller (Sutter P-97, Sutter Instrument, Novato, CA, USA) and a glass capillary needle (3.5′′, Drummond). GenePharma designed and synthesized a small interfering RNA targeting the open reading frame (ORF) of *CYP4H31* (si-*CYP4H31*) (Table 2). For the microinjection of miRNA, DR strain female adult mosquitoes were collected within 12 h PE and frozen at − 20 °C for 3−5 min. These mosquitoes were divided into three groups and injected in the thorax with different moieties. The negative control group (NC1) was injected with 0.5 μl of miRNA control mimic at a dose of 20 nmol/l, and the experimental group (miR-4448 mimic) was injected with 0.5 μl of the miR-4448 mimic under the same conditions at a final dose of 20 nmol/l. For the microinjection of siRNA, the negative control group (NC2) was injected with 69 nl of control at a dose of 5 μg/μl, and the experiment group (si-*CYP4H31*) was injected with 69 nl of si-*CYP4H31* under the same conditions at a final dose of 5 μg/μl. Thereafter, the mosquitoes were transferred to holding tubes and maintained in our laboratory with a constant light/dark cycle (14:10 h) at 28 °C with 70–80% humidity. After 72 h, the expression levels of miR-4448 and its target gene *CYP4H31* were validated using qRT-PCR. Three biological replicates with three technical replicates, each replicate containing 20 female mosquitoes, were performed.

### Eukaryotic expression vector pIB/V5-His construction, cell culture, and transfection

Standard molecular biology procedures were used for plasmid construction [27]. Overlap PCR was performed to amplify the ORF of *CYP4H31* using corresponding primer pairs (Table 1) from *Cx. quinquefasciatus*, which was inserted between unique restriction enzyme sites (*SpeI*/*XhoI*) of the eukaryotic expression vector, pIB/V5-His (Invitrogen). The positive recombinant plasmid, named pIB/V5-His-CYP4H31, was confirmed using DNA sequencing.

*Aedes albopictus* C6/36 cells (CRL-1660; ATCC, Manassas, VA, USA) were cultured in DMEM supplemented with 10% (v/v) FCS. The C6/36 cells were grown in a 6-well plate at 28 °C in a 5% CO_2_-humidified incubator at. The cells were then plated at 5 × 10^5^ cells/well and incubated for 24 h in a 6-well plate in 2.5 ml of complete growth medium. The cells were transfected when they reached 60–80% confluence. The transfection protocol was as follows: the plasmid DNA (pIB/V5-His-*CYP4H31*) was diluted to 1.5 ng per 100 µl in complete growth medium followed by the addition of 5 µl of FuGENE HD transfection reagent. The DNA mixture was incubated at room temperature for 25 min and then added to the medium below the surface. The plate was rocked back and forth and from side to side to ensure distribution over the entire plate surface. Meanwhile, C6/36 cells transfected with pIB/V5-His were used as controls. Three biological replicates with three technical replicates were performed.

### qRT-PCR and Western blotting analysis of CYP4H31 in the transfection cells

At 48 h after transfection, the transiently transfected C6/36 cells were subjected to western blotting and qRT-PCR. To evaluate the *CYP4H31* transfection efficiency, total RNA was isolated from the transfected cells, and qRT-PCR was performed, as described above, to check the expression level of *CYP4H31*.

Transfected cells were washed with phosphate-buffered saline (PBS). Protein was extracted from the cells after digestion with trypsin solution and lysis using radioimmunoprecipitation assay (RIPA) buffer (Beyotime, Shanghai, China). Protein concentrations were tested using a bicinchoninic acid (BCA) Protein Assay kit (Pierce, Rockford, IL, USA). Soluble protein (50 µg) was denatured and subjected to 10% SDS-PAGE. Proteins were transferred to a nitrocellulose membrane using Trans-Blot SD Cell and Systems for 60 min at 300 mA (Bio-Rad, Hercules, CA, USA). The membrane was washed twice in 1 × Tris-buffered saline-Tween 20 (TBS-T), and then blocked for 60 min at 37 °C in 5% Difco™ Skim Milk (BD Biosciences, San Jose, CA, USA). The membrane was incubated with anti-His-Tag monoclonal primary antibodies (1:1000, NovaGen, Madison, WI, USA) and β-actin monoclonal primary antibodies (1:2000, ABGENT, Suzhou, China), with shaking overnight at 4 °C. The membranes were then washed with TBS-T and incubated with horseradish peroxidase (HRP)-conjugated goat anti-mouse secondary antibodies (1:2000, Bioworld, Shenzen, China) in blocking buffer at 37 °C for 2 h. The membranes were washed thoroughly with TBS-T before imaging using BIO-RAD UNIVERSAL HOOD II and Pierce™ ECL Western Blotting Substrate, according to the manufacturer's instructions.

### Cell viability assay using Cell Counting Kit-8

The *CYP4H31* ORF (GenBank: KM056314.1) of *Cx. pipiens pallens* was amplified, inserted into vector pIB/V5-His, and transfected into C6/36 cells. Total RNA and protein were extracted from C6/36 cells of blank group (WT), the pIB/V5-His control group (NC), and the experimental group (pIB/V5-His-*CYP4H31*). *CYP4H31* overexpression in relation to deltamethrin resistance was assessed by measuring cell viability using Cell Counting Kit-8 (CCK-8; Dojindo, Japan) [28]. Cells (100 µl) were added to each well of a 96-well plate 5 × 10^3^ cells/well and incubated in a 5% CO_2_-humidified incubator at 28 °C for 24 h. Then, the cells were treated with various concentrations of deltamethrin in 100 µl (0, 10^0.5^, 10^1^, 10^1.5^, 10^2^, and 10^2.5^ mg/l) [29]. Twenty-four hours later, CCK-8 solution (10 µl) was added to the wells and incubated for 28 °C for 3 h. The absorbance was then detected using dual wavelength spectrophotometry at 450 nm and 630 nm in a microplate reader. Dimethyl sulfoxide (DMSO, Sigma, St, Louis, MO, USA) was used to dissolve deltamethrin and the final concentration of DMSO was 0.5% (v/v) for the different concentrations of deltamethrin [22]. Three biological replicates with three technical replicates were performed.

### American CDC bottle bioassay

According to published guidelines, American Centers for Disease Control and Prevention (CDC) bottle bioassays were conducted to detect the sensitivity of mosquitoes injected with the miR-4448 mimic and si-*CYP4H31* to deltamethrin [30]. Each 250-ml bottle and its cap were coated with 1 ml of deltamethrin solution using inversion and rolling of the bottles. Control bottles were coated using 1 ml of acetone. A sheet was used to cover all bottles, which were left to dry in the dark. Twenty mosquitoes were placed in each bottle and exposed to deltamethrin or acetone for 120 min. Following exposure, mosquitoes subjected to *CYP4H31* knockdown were monitored at 15-min intervals up to 2 h. Mosquitoes were considered dead if they could no longer stand [31]. The percent mortality (*y*-axis) was plotted against time (*x*-axis) using a linear scale.

### Statistical analyses

Statistically significant qualitative variables were detected using the GraphPad Prism 8.0 software (GraphPad Inc., La Jolla, CA, USA), and statistical significance was accepted at *P* < 0.05. Qualitative variables were analyzed using the Chi-square test, while quantitative variables were assessed using analysis of variance [32, 33].

## Results

### MiR-4448 targets *CYP4H31*

**Fig. 1:**
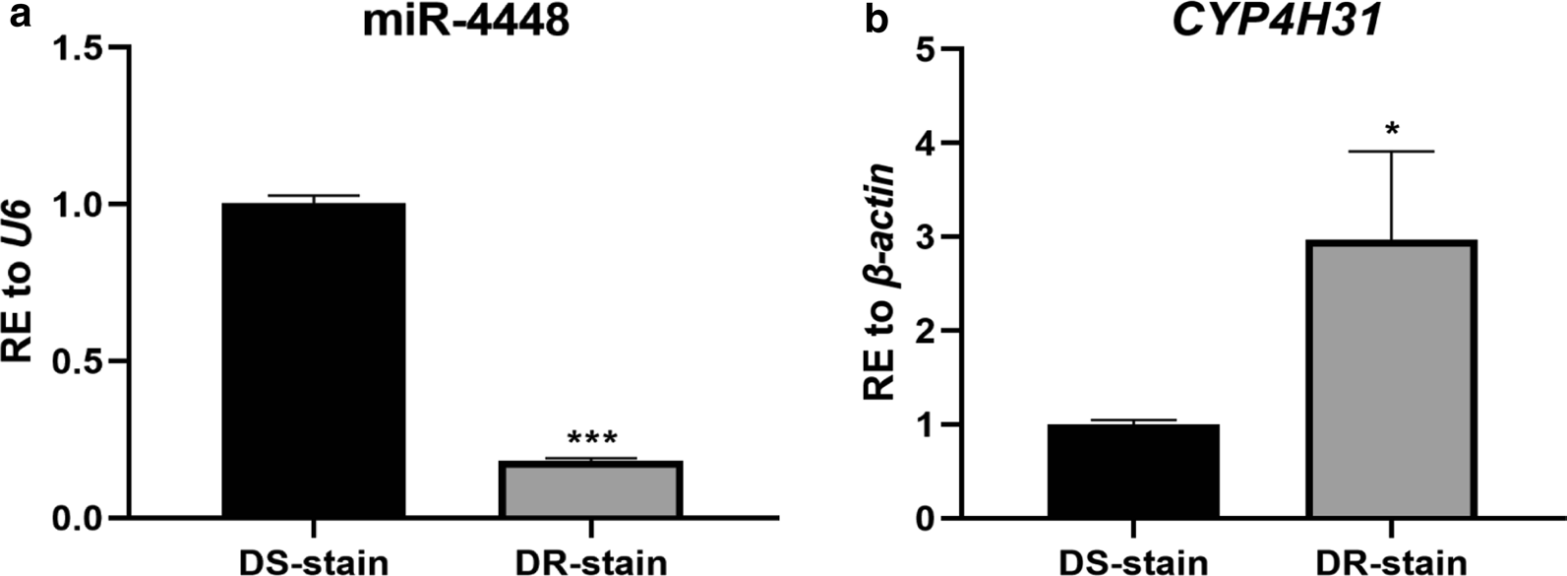
Detecting the expression level of miR-4448 and *CYP4H31* in mosquitoes using qRT-PCR. The expression of miR-4448 was lower and that of *CYP4H31* was higher in the DR strain than that in the DS strain. **a** The expression of miR-4448 was 6.49-fold higher in the DS strain than in the DR strain. **b** The expression of *CYP4H31* was 2.77-fold higher in the DR strain than in the DS strain. Data are representative of three technical replicates of three biological replicates and are indicated as the mean ± SE; *P < 0.05, ***P < 0.001. *RE* relative expression, *qRT-PCR* quantitative real-time reverse transcription PCR, *DR-strain* deltamethrin-resistant strain, *DS-strain* deltamethrin-susceptible strain

**Fig. 2:**
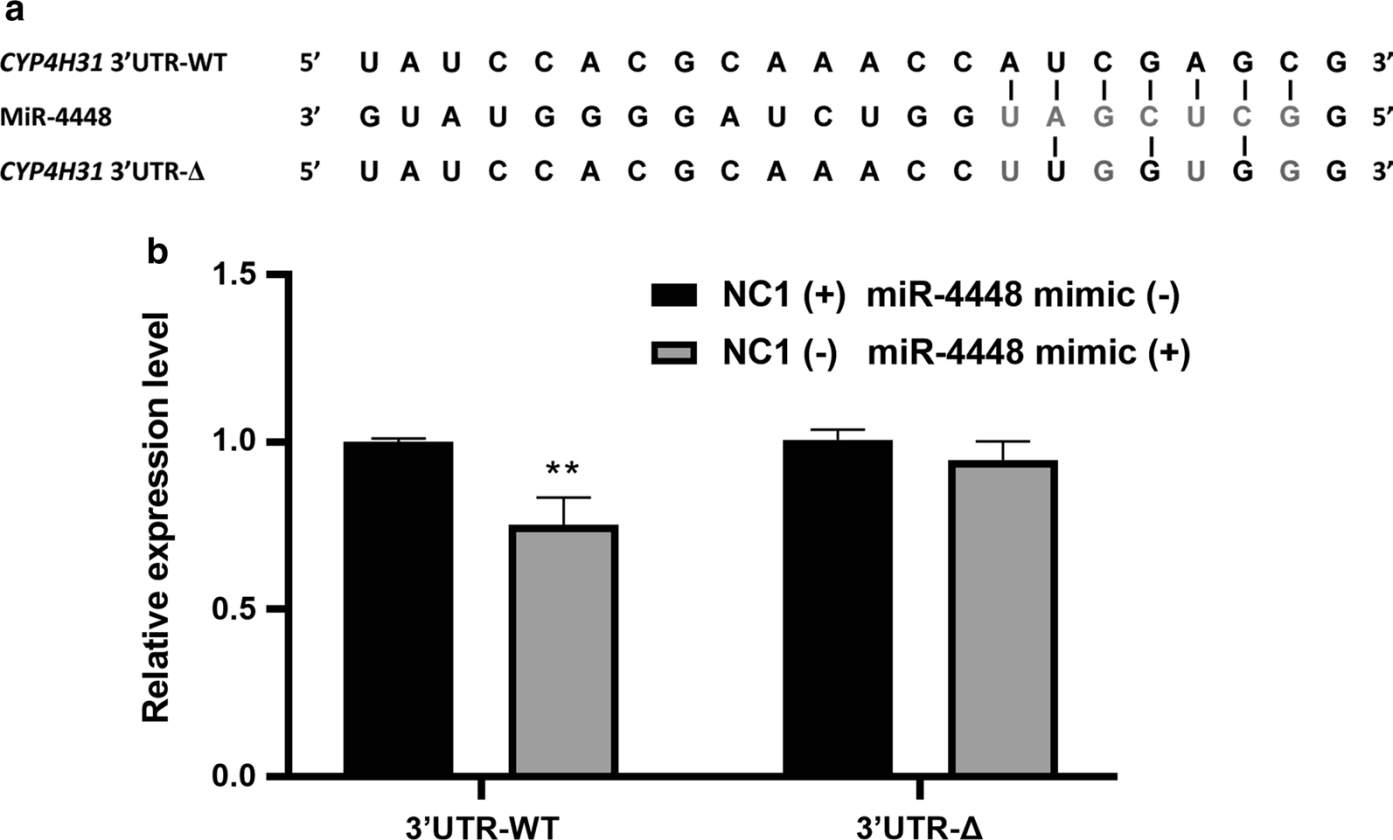
Dual-luciferase report assays were used to determine the interaction between miR-4448 and *CYP4H3*1 *in vitro*. MiR-4448 directly regulated *CYP4H31* expression through 3′ UTR sites. **a** The predicted *CYP4H31* 3′ UTR miR-4448 binding site. **b** *CYP4H31* assessed using a dual-luciferase reporter assay. The luciferase intensity was reduced by 24.67%. Data are representative of three technical replicates of three biological replicates and are indicated as the mean ± SE; **P < 0.01. *RE* relative expression, *3′ UTR* 3′ untranslated region

Preliminary Solexa sequencing results showed that the expression of miR-4448 was significantly different between the DS and DR strains [22]. In this study, miR-4448 showed 6.49-fold higher expression in the DS strain compared with that in the DR strain (Fig. 1a, ***P < 0.001), while the predicted target gene, *CYP4H31*, showed 2.77-fold lower expression in the DS strain than that in the DR strain (Fig. 1b, *P < 0.05). The contrasting expression patterns suggested that *CYP4H31* might be the target gene of miR-4448. Dual-luciferase report assays were then used to determine the interaction between miR-4448 and *CYP4H31* *in vitro*. Plasmids inserted with the 3′ UTR-WT or 3′ UTR-Δ of *CYP4H31* along with the control plasmid, pGL4.7, were cotransfected into HEK 293-T cells and then treated with the miRNA-4448 mimic or miRNA negative control (NC1). The results showed that miR-4448 treatment inhibited the luciferase activity from the WT 3′ UTR construct markedly (by 24.67%), while no significant change occurred when the cells were treated with the negative control. Meanwhile, no increase in luciferase activity was observed in the 3′ UTR-Δ group when treated with the miR-4448 mimic (Fig. 2b, **P < 0.01). Therefore, *CYP4H31* was verified as a target of miR-4448 *in vitro*.

### MiR-4448 modulates mosquito deltamethrin resistance

**Fig. 3:**
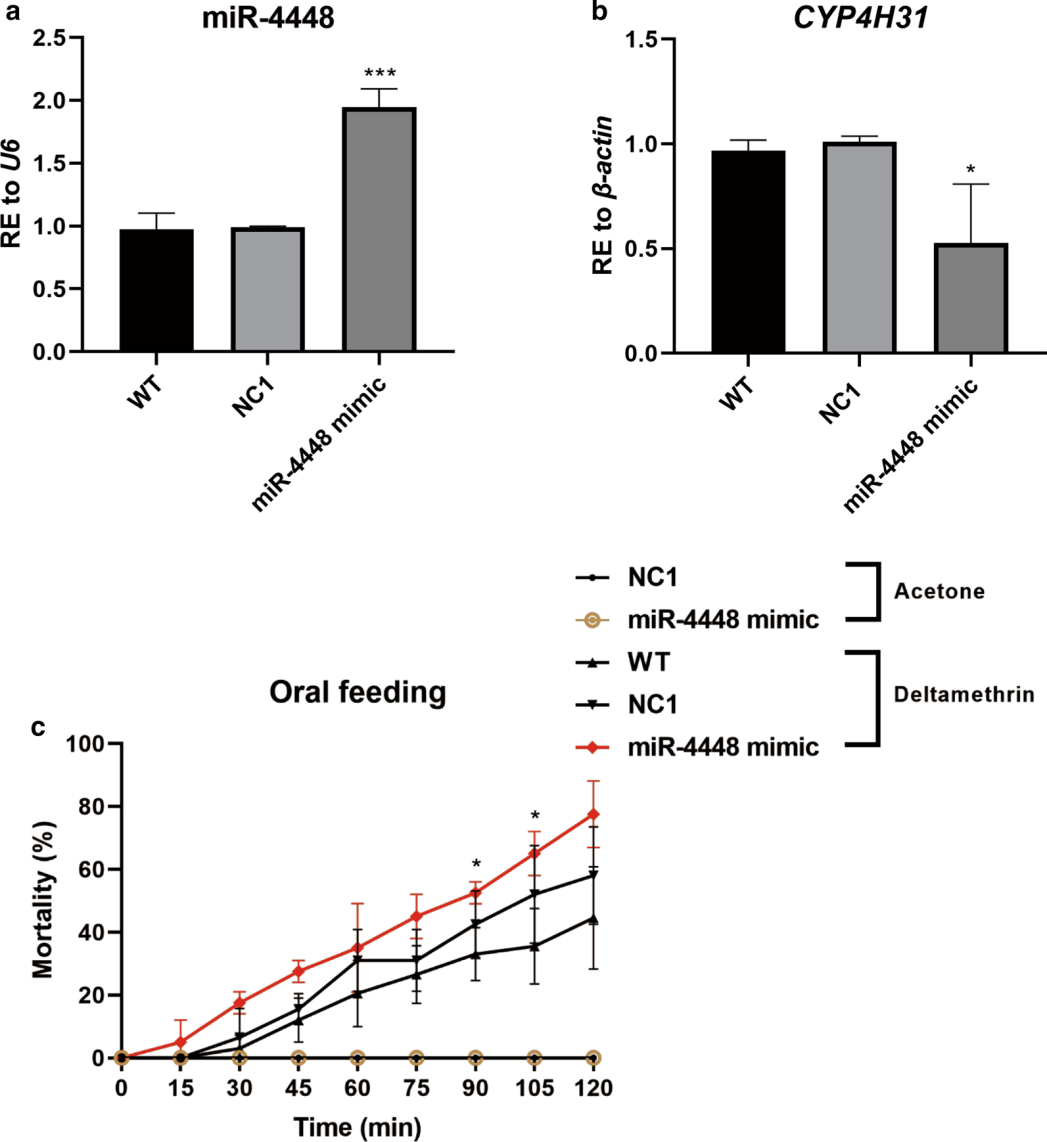
CDC bottle assays were used to detect the function of miR-4448 by oral feeding of the miR-4448 mimic in DR strain mosquitoes. Oral feeding with the miR-4448 mimic could reduce mosquito resistance to deltamethrin. **a**, **b** Oral feeding of the miR-4448 mimic upregulated the expression of miR-4448 (by 1.97-fold) and downregulated the expression of *CYP4H31* (47.85%) in DR strain. **c** Mosquito mortality was assessed after incubation for a 2 h in a CDC bottle containing 7 mg/l deltamethrin. The mortality of the miR-4448 mimic group was higher than that of the acetone control, WT, and miRNA control mimic (NC1) groups. At 90 and 105 min, the mortality rates of miR-4448 mimic-fed mosquitoes were 52.5% (21/40) and 65.0% (26/40), respectively, which were higher than those of the NC1 group (42.4% [14/33] and 51.5% [17/33]). Data are representative of three technical replicates of three biological replicates and are indicated as the mean ± SE; ***P < 0.001, *P < 0.05. *RE* relative expression, *DR-strain* deltamethrin-resistant strain

**Fig. 4:**
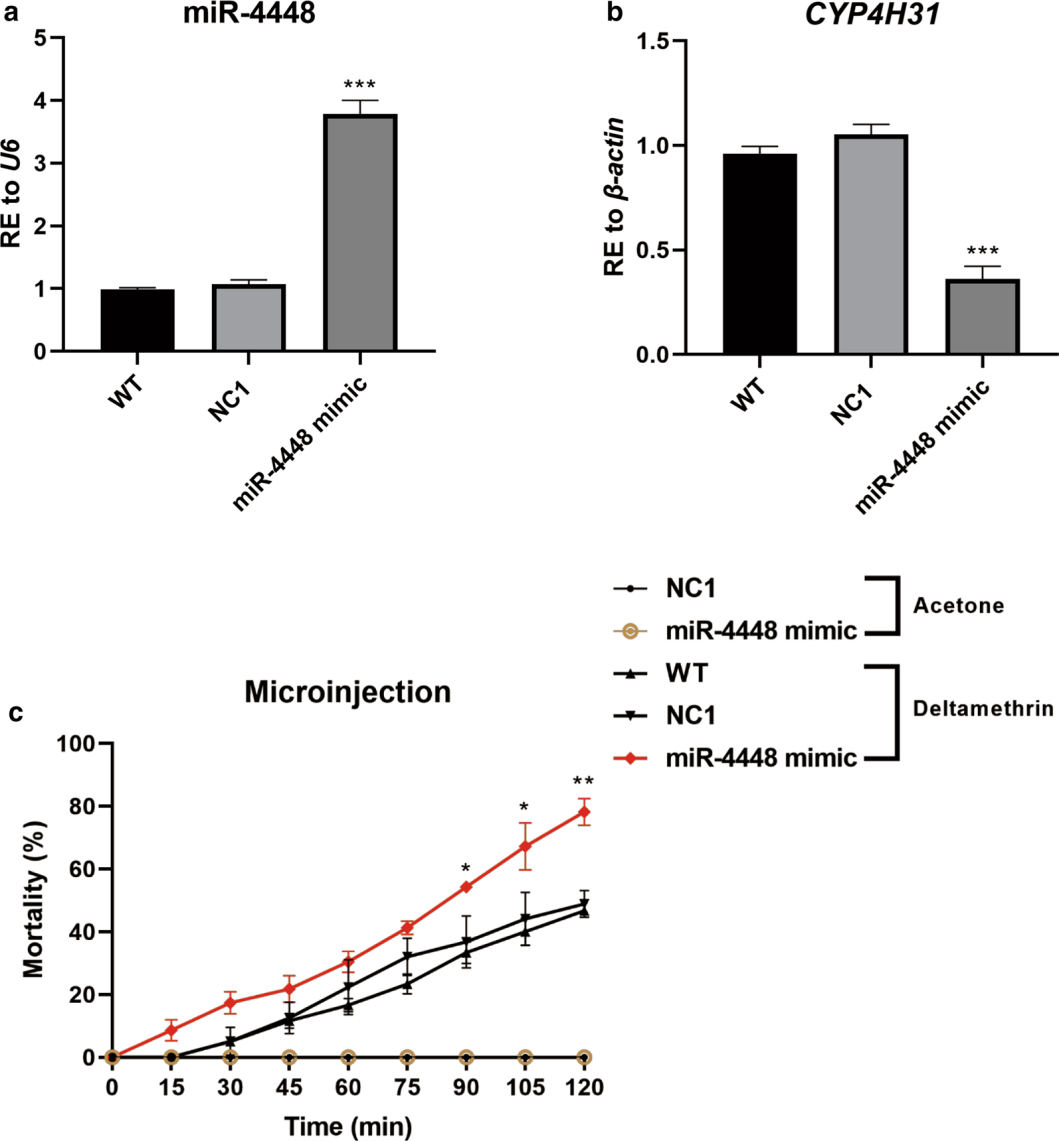
CDC bottle assays were was used to detect the function of miR-4448 by microinjection of the miR-4448 mimic in DR strain mosquitoes. Microinjection of miR-4448 mimic could reduce mosquito resistance to deltamethrin. **a**, **b** Microinjection of the miR-4448 mimic upregulated the expression of miR-4448 (by 3.53-fold) and downregulated the expression of *CYP4H31* (65.82%) in the DR strain. **c** Mosquito mortality was assessed after incubation for a 2 h in a CDC bottle containing 7 mg/l deltamethrin. The mortality of the miR-4448 mimic group was higher than that of the acetone control, WT, and miRNA control mimic (NC1) groups. At 105 min, in the mimic group, the mortality rate was 67.4% (31/46); in the NC1 group, the mortality rate was 43.9% (18/41); in the WT group, the mortality rate was 40.0% (24/60). At 120 min, in the mimic group, the mortality rate was 78.3% (36/46), while it was 48.8% (20/41) in the NC1 group and 46.7% (28/60) in the WT group. Data are representative of three technical replicates of three biological replicates and are indicated as the mean ± SE; ***P < 0.001, **P < 0.01, *P < 0.05. *RE* relative expression, *DR-strain* deltamethrin-resistant strain

To determine whether miR-4448 could regulate deltamethrin resistance in mosquitoes, the miR-4448 mimic or miRNA mimic control was supplied to DR strain mosquitoes via oral feeding. The relative miR-4448 expression was 1.97-fold higher in the mosquitoes fed with the miR-4448 mimic compared with those in the NC1 control group (Fig. 3a, ***P < 0.001), which suggested that in the DR strain, miR-4448 was successfully overexpressed. In these cells, the transcription level of *CYP4H31* was decreased about 47.85% (Fig. 3b, *P < 0.05), which suggested that *CYP4H31* is a direct *in vivo* target of miR-4448. In the CDC bottle bioassay, the group fed with the miR-4448 mimic had a higher mortality rate compared with those in the NC1 groups. At 90 and 105 min, the mortality rate of miR-4448 mimic-fed mosquitoes was 52.5% (21/40) and 65.0% (26/40), respectively, which was higher than those in the NC1 groups (42.4% [14/33] and 51.5% [17/33]) (Fig. 3c, *P < 0.05). To further validate the results obtained by oral feeding, we injected the miR-4448 mimic or miRNA mimic control into each mosquito at 12 h PE. The microinjection results showed the efficient overexpression of miR-4448 (by 3.53-fold) and the significantly decreased expression level of *CYP4H31* (65.82%) in the miR-4448 mimic injection group (Fig. 4a, b, ***P < 0.001). The expression trend was consistent with the results of the oral feeding experiment. Additionally, the results of the CDC bottle bioassay showed significantly higher mortality rates after injecting miR-4448 mimic compared with the control. At 105 min, in the mimic group, the mortality rate was 67.4% (31/46); in the NC1 group, the mortality rate was 43.9% (18/41); in the WT group, the mortality rate was 40.0% (24/60). Furthermore, at 120 min, in the mimic group, the mortality rate was 78.3% (36/46), while it was 48.8% (20/41) in the NC1 group and 46.7% (28/60) in the WT group (Fig. 4c, **P < 0.01, *P < 0.05). Taken together, oral feeding and microinjection both demonstrated that miR-4448 could modulate deltamethrin resistance of mosquitoes by downregulating the expression of *CYP4H31*.

### *CYP4H31* functions in mosquito deltamethrin resistance

**Fig. 5:**
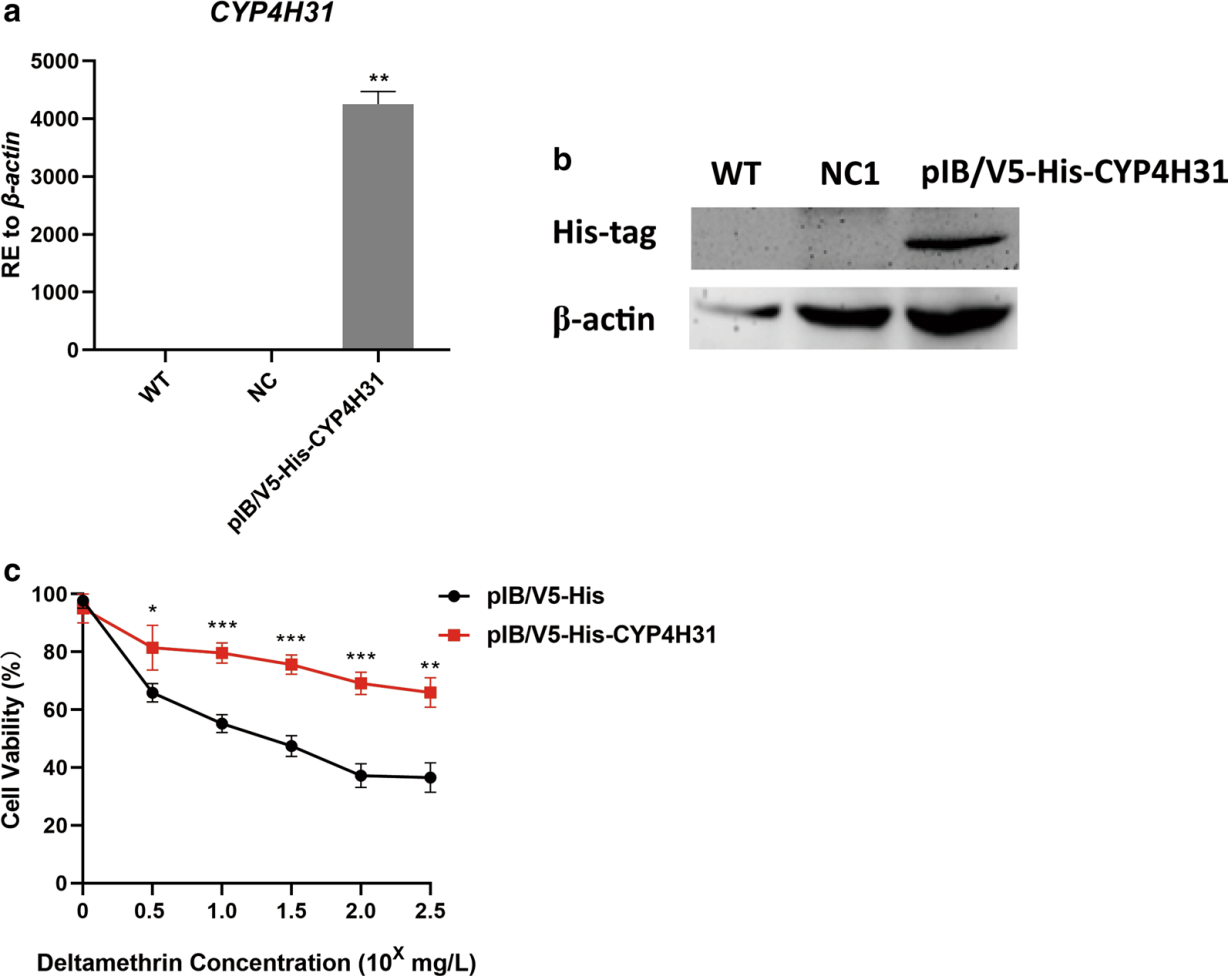
CCK-8 assays were employed to detect cell viability after deltamethrin treatment. Overexpression of *CYP4H31* upregulated the cell viability of C6/36 cells under deltamethrin stress. **a** qRT-PCR analysis of *CYP4H31* in transfected C6/36 cells. The cells transfected with pIB/V5-His-*CYP4H31* showed a significantly higher transcription level (851.0-fold). **b** Western blotting experiments using His-tag antibodies to detect the CYP4H31 protein. **c** The pIB/V5-His-CYP4H31 construct-transfected C6/36 cells were treated with deltamethrin and their viability was calculated using a CCK-8 kit. The viability of pIB/V5-His-CYP4H31-transfected cells was higher than that in the pIB/V5-His group. Data are representative of three technical replicates of three biological replicates and are indicated as the mean ± SE; ***P < 0.001, **P < 0.01, *P < 0.05. *RE* relative expression

To determine the function of *CYP4H31* in mosquito deltamethrin resistance, transient transfection assays were performed in C6/36 cells *in vitro*, and their sensitivity to deltamethrin was determined after transfection. The results showed the expression level of *CYP4H31* was 851.0-fold higher in the experimental group than in the NC group (Fig. 5a, **P < 0.01). Western blotting demonstrated that the band could be detected using anti-His-tag antibodies in the pIB/V5-His-*CYP4H31* group (Fig. 5b). Thus, transcript and protein level detection proved the transfection was successful. To investigative the sensitivity of the transiently transfected C6/36 cells to deltamethrin, a CCK-8 kit was employed to detect cell viability after deltamethrin treatment. The percentage of viable cells among those transfected with pIB/V5-His-*CYP4H31* was significantly higher than those in the NC and WT groups (Fig. 5c, ***P < 0.001, **P < 0.01, *P < 0.05). The data showed that *CYP4H31* could increase mosquito cell resistance to deltamethrin.

**Fig. 6:**
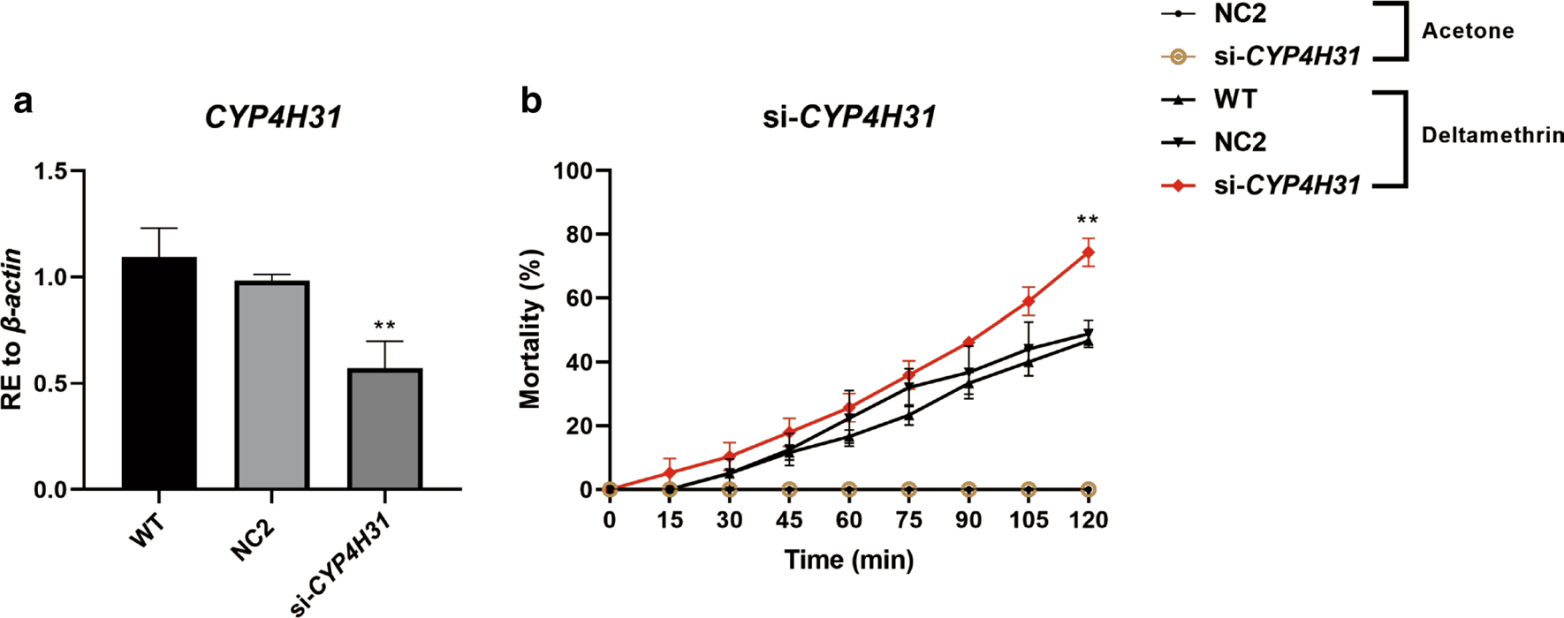
*CYP4H31* RNAi (si-*CYP4H31*) in DR strain mosquitoes. Microinjection of an siRNA targeting *CYP4H31* in female adult mosquitoes reduced their resistance to deltamethrin. **a** Microinjection of si-*CYP4H31* downregulated the expression of *CYP4H31* (41.90%) in the DR stain. **b** Mosquito mortality was assessed after incubation for a 2 h in a CDC bottle containing 7 mg/l deltamethrin. The mortality of the si-*CYP4H31* group was higher than that in the acetone control, WT, and control siRNA (NC2) groups. At 120 min, the mortality rate was 74.3% (29/39) in si-*CYP4H31*-injected mosquitoes, while it was 46.7% (21/45) in the NC2 group and 48.7% (20/41) in the WT group. Data are representative of three technical replicates of three biological replicates and are indicated as the mean ± SE; **P < 0.01. *RE* relative expression, *DR-strain* deltamethrin-resistant strain

To further evaluate whether *CYP4H31* participates in mosquito resistance to deltamethrin *in vivo*, we conducted phenotypic experiments using *CYP4H31* RNAi knockdown (si-*CYP4H31*) in DR strain mosquitoes. We expected that the RNAi-mediated ablation of the physiologically relevant target of miR-4448 would display the same phenotype as that caused by miR-4448 overexpression. *CYP4H31* expression decreased by 41.90% in the si-*CYP4H31* injection group compared with that in the negative control group (NC2) (Fig. 6a, **P < 0.01). RNAi silencing of *CYP4H31* in mosquitoes resulted in increased sensitivity to deltamethrin. At 120 min, the mortality rate was 74.3% (29/39) in si-*CYP4H31*-injected mosquitoes, while it was 46.7% (21/45) in the NC2 group and 48.7% (20/41) in the WT group (Fig. 6b, **P < 0.01). These results suggested that *CYP4H31* does indeed play a role in mosquito deltamethrin resistance.

## Discussion

Our results showed downregulation of miR-4448 in the DR strain, which suggested that miR-4448 might be involved in the regulation of deltamethrin resistance in *Cx. pipiens pallens*. Multiple approaches to miRNA target prediction were used to identify the physiologically relevant miR-4448 target contributing to the miR-4448-mimic phenotypes. We identified *CYP4H31* as a direct target of miR-4448 *in vitro* and *in vivo*. The results from bioinformatic predictions showed that miR-4448 might directly regulate the expression of the P450 gene, *CYP4H31*. A dual-luciferase reporter assay using a luciferase reporter vector containing *Cx. pipiens pallens CYP4H31* 3′ UTR cotransfected with the miR-4448 mimic resulted in a decrease in *Renilla* luciferase activity *in vitro*. Overexpression of miR-4448 by oral feeding and microinjection of an miR-4448 mimic reduced *CYP4H31* expression and increased the mosquitoes’ sensitivity to deltamethrin *in vivo*. By contrast, *CYP4H31* overexpression decreased mosquito cell sensitivity to deltamethrin, and intrathoracic microinjection of dsRNA of *CYP4H31* (si-*CYP4H31*) increased the mosquitoes’ sensitivity to deltamethrin. Taken together, these results further confirmed *CYP4H31* as an authentic miR-4448 target and indicated that miR-4448 might participate in deltamethrin-resistance by regulating *CYP4H31* in mosquitoes.

MicroRNAs (~ 23 nt) are endogenous RNAs that play an important gene-regulatory role by pairing with the 3′ UTR of protein-coding gene mRNAs to direct their posttranscriptional repression [34]. Dysregulation of miRNAs has been reported in host-pathogen interactions, metabolism, development, and insecticide resistance [21]. Previously, our group performed Solexa high-throughput sequencing and showed that miR-4448 was highly expressed in DS strain mosquitoes [22]. In this study, to further investigate the function of miR-4448 in deltamethrin-resistant mosquitoes, we first identified that the pre-miR-4448 sequence was present in *Cx. pipiens pallens*. The qRT-PCR results showed that the conserved miRNA, miR-4448, was enriched in the DS strain mosquitoes.

Many studies have shown that multiple, complex resistance mechanisms, particularly increased metabolic detoxification of insecticides, are likely to be responsible for insecticide resistance (reviewed in [16]). Commonly, metabolic detoxification, especially by CYPs, is the main molecular mechanism of insecticide resistance [35]. The overproduction of CYPs in resistant populations could, in principle, lead to a negative cross-resistance between different insecticides in insects, in which detoxification of one insecticide (for example pyrethroids) occurs at the same time as activation of another pro-insecticide (such as organophosphates, ketoenols, or clorfenapyr) [36]. Recently, researchers reported that miRNAs could mediate insecticide resistance through *CYP* genes [37–39]. In the present study, *CYP4H31* was identified as a direct *in vitro* and *in vivo* target of miR-4448. A dual-luciferase reporter assay comprising a *CYP4H31* 3′ UTR-containing luciferase reporter vector, which was cotransfected together with the miR-4448 mimic, produced decreased *in vitro Renilla* luciferase activity. Meanwhile, microinjection of the miR-4448 mimic decreased the *CYP4H31* transcript level *in vivo*, which further confirmed that in mosquitoes, *CYP4H31* is a target gene of miR-4448. *CYP4H31* belongs to the CYP4 family as a member of the monooxygenase cytochrome P450 (CYPs) superfamily [40]. CYP4 family genes were proposed as the most important *P450* genes involved in pyrethroid resistance in *Anopheles sinensis* [41].

Next, using oral feeding and RNAi technology, in combination with the CDC bottle assay, the present study made significant progress toward determining the regulatory role of miRNAs in insecticide resistance. In mosquitoes, miR-4448 function decreases deltamethrin resistance by inhibiting *CYP4H31* expression. However, the mortality of miR-4448-mimic-supplied mosquitoes showed no significant change compared with the controls, possibly because we could not control the amount of microRNA mimic taken up by each mosquito. To date, novel strategies, including transgenic plants, engineered microorganisms, and nano-scale formulations, have been developed to improve the efficacy of miRNA; however, many hurdles must be overcome before this technology becomes a reliable method of pest management [42]. Notwithstanding, in our study, the miR-4448-mimic-injected mosquitoes displayed drastically higher sensitivity to deltamethrin and resulted in significantly increased mortality in the DR strain. Furthermore, our study showed that high expression of *CYP4H31* could increase resistance to deltamethrin and consequently improve cell viability. In contrast, low expression of *CYP4H31* after miR-4448 mimic oral feeding or microinjection, and *CYP4H31* RNAi, resulted in increased sensitivity to deltamethrin and higher mortality. Taken together, our findings demonstrated that *CYP4H31* is related to mosquito deltamethrin resistance.

## Conclusion

**Fig. 7:**
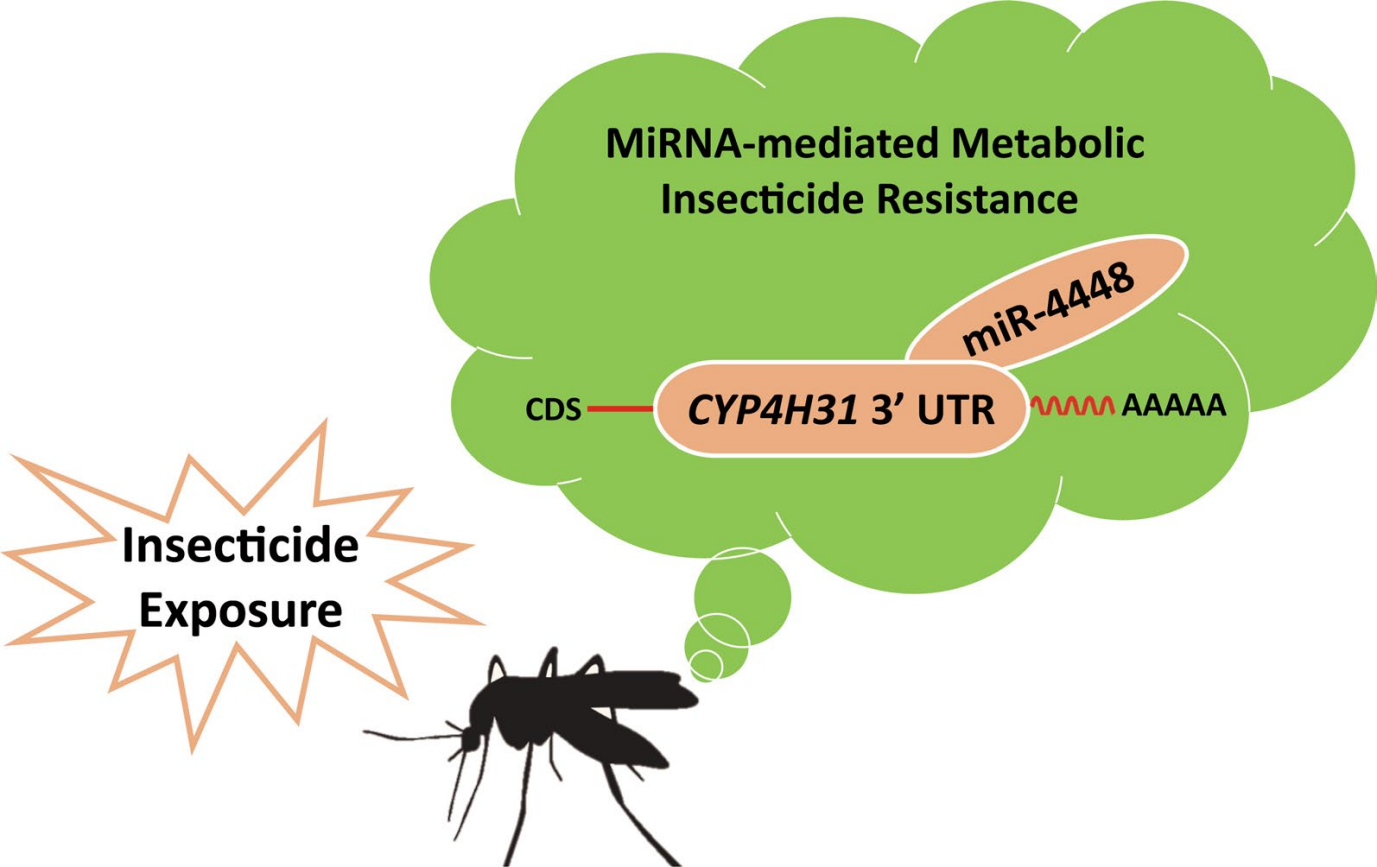
The regulatory map of miR-4448′s involvement in deltamethrin resistance by targeting *CYP4H31* in *Culex pipiens pallens*

Our study has established a fundamental role for miR-4448 in the regulation of mosquito deltamethrin resistance through its target, *CYP4H31*, in mosquitoes (Fig. 7). Further investigation of *CYP4H31*, e.g. using gene editing, is warranted to determine its exact function in deltamethrin resistance. Our findings revealed a mechanism of insecticide resistance, which could lead to new methods to control mosquito populations.

## Data Availability

Data supporting the conclusions of this article are included within the article. All data are fully available without restriction upon request.

## Abbreviations

miRNA: MicroRNA
DR strain: Deltamethrin-resistant strain
DS strain: Deltamethrin-susceptible strain
qRT-PCR: Quantitative real-time reverse transcription PCR
gDNA: Genomic DNA
3′-UTR: 3′ Untranslated region
3 d PE: 3 days post-eclosion
CCK-8: Cell Counting Kit-8
